# Repeat resection for recurrent glioblastoma – does timing matter?

**DOI:** 10.1007/s11060-026-05477-8

**Published:** 2026-02-24

**Authors:** Obada T. Alhalabi, Kirill Mironov, Khurshed Nabiev, Johanna Krämer, Nour Gareib, Henri Olldashi, Stefan Joser, Marianne Schell, Sandro M. Krieg, Andreas W. Unterberg, Christine Jungk

**Affiliations:** 1 Department of Neurosurgery, Heidelberg University Hospital, Im Neuenheimer Feld 400, 69120 Heidelberg, Germany; 2 Medical Faculty, University Heidelberg, Heidelberg, Germany; 3 Clinical Cooperation Unit Neurooncology, German Cancer Consortium (DKTK), German Cancer Research Center (DKFZ), Heidelberg, Germany; 4 Department of Neuroradiology, University Hospital Heidelberg, Heidelberg, Germany; 5 Division for Computational Neuroimaging, Heidelberg University Hospital, Heidelberg, Germany

**Keywords:** Recurrent glioblastoma, Repeat resection, Volumetric analysis, Pseudo-progression, Timing of surgery

## Abstract

**Background:**

The optimal timing of repeat surgical resection in patients with recurrent IDH-wildtype glioblastoma (rGB) remains unclear. We aimed to characterize temporal patterns between radiological suspicion of recurrence and repeat resection and to evaluate the impact of early versus delayed surgery on the extent of resection (EOR), functional outcomes, adjuvant therapy, and survival

**Methods:**

We retrospectively analyzed a consecutive cohort of 150 patients who underwent resection for histopathologically confirmed rGB between 2015 and 2023 at a single tertiary care center. Assessment of contrast-enhancing preoperative and residual tumor volumes (RTV on early postoperative MRI) was performed using semi-automated segmentation. Based on the mean or median time between suspicion of recurrence and repeat resection, patients were stratified into early and late surgery groups. RANO Resect criteria and a 0.175-ml RTV threshold were used to classify EOR. Functional outcomes, postoperative treatment, as well as progression-free survival (PFS) after repeat resection, and overall survival (OS) after suspicion of recurrence were compared between groups.

**Results:**

Mean and median time from suspicion of recurrence to repeat resection were 54 and 24 days, respectively, with 75% of patients undergoing reoperation within 6 weeks. Applying the mean cut-off, early (n=120) and late (n=30) surgery groups showed comparable baseline demographics, performance status, tumor eloquence, and preoperative neurological deficits. Preoperative tumor volumes were significantly smaller in the early surgery group (12.7 vs. 25.9 ml, p=0.002). Late surgery was associated with a trend toward higher RTV and lower rates of gross total resection, though without statistical significance. Rates of transient and permanent postoperative neurological deficits were low (15% and 2%) and did not differ between groups. Adjuvant treatment patterns differed, with early surgery patients more frequently receiving CCNU-based chemotherapy, while late surgery patients more often received no further treatment. Median OS after suspicion of recurrence (12.4 vs 14.3 months) and PFS after repeat resection (4 months in both groups) were not significantly different between early and late surgery groups. A re-analysis using the median of 24 days as cut-off revealed similar results with regards to survival and functional outcomes.

**Conclusion:**

Most patients with repeat resections for rGB underwent surgery shortly after radiological suspicion of recurrence. While delayed surgery was associated with larger tumor volumes and a trend toward less favorable EOR and adjuvant treatment, timing of surgery alone was not associated with functional outcomes or survival. These findings support individualized decision-making for repeat resection based on clinical and radiological factors rather than timing alone.

## Introduction

Glioblastoma (GB), isocitrate dehydrogenase (IDH) wildtype is characterized by therapy-resistance and poor prognosis [1, 2]. Indeed, the current median overall survival (OS) is around 15 months [3, 4]. Despite multimodal therapy, tumor recurrence is almost inevitable with a limited repertoire of standard-of-care therapy options at recurrence [5]. Many trials involving systemic therapy for recurrent GB (rGB) have failed in the past [6, 7]. At the same time, several studies have shown the clinical benefit of repeat resection for selected patients dependent on tumor location and functional status [8–10].

The importance of maximizing the extent of resection (EOR) at recurrence has been well established [11–14]. The median survival after repeat resection is reported to be at 12 to 18 months [15–17]. This survival is achieved under a reasonable rate of non-resolving post-operative neurological deterioration of about 8% in previous cohorts [9, 14, 18–21].

Reflecting the challenges in the assessment of treatment response, up to 40% of GB patients receiving chemoradiotherapy develop increased contrast enhancement (CE) on magnetic resonance imaging (MRI) during treatment, which mostly stabilizes or even spontaneously regresses [22, 23]. This putative pseudo-progression occurs at 3 to 6 months after initial therapy [24, 25] and can be associated with clinical deterioration [26]. It is unknown how long such imaging changes can be tolerated before anti-tumor treatment should be initiated and once initiated, how treatment response should be assessed before repeat resection is triggered.

Consequently, therapeutic decision-making at recurrence remains highly heterogeneous. While some patients undergo upfront repeat resection, others are managed with a watch-and-wait strategy or receive salvage systemic treatment or radiotherapy. In the latter group, treatment responses are variable, with some patients showing early progression, and others achieving initial disease control followed by delayed progression. Consequently, the optimal timing of repeat resection and possible pitfalls of delayed repeat surgery for recurrent glioblastoma remain uncertain.

In particular, it is unclear whether a watch-and-wait strategy allows tumors to progress to a size or location that limits resectability, increases surgical risk, or adversely affects neurological outcomes and survival. Therefore, this study aimed to evaluate whether delayed repeat resection is associated with a higher risk of postoperative deficits or differences in oncological outcomes compared with earlier repeat resection at suspicion of GB recurrence.

## Methods

### Study population

This study analyzed a consecutive cohort of patients with repeat resection for local recurrence of a previously resected IDH-wildtype glioblastoma (as per WHO 2021 classification [27]) treated between 2015 and 2023 at the Department of Neurosurgery, University Hospital Heidelberg that was retrospectively identified from our institutional database. Only patients with histopathologically diagnosed GB recurrence were included, while histopathologically confirmed radiation-induced necroses were excluded. Inclusion required the availability of preoperative MRI (3 Tesla; Siemens Magnetom Verio™, Prisma™, or Skyra™) and early postoperative MRI (epMRI) acquired within 48 hours after surgery using comparable scanner specifications (Siemens, 3 Tesla). Radiological evaluation of tumor recurrence followed the RANO (Response Assessment in Neuro-Oncology) criteria [13]. Eligibility for repeat resection at our center required sufficient performance status, surgically accessible tumor location, and multidisciplinary consensus that repeat resection was clinically appropriate. All patients underwent tumor resection with intraoperative MRI (1.5T Siemens Espree).

### Timing of surgery

For stratification into “early” and “late” repeat resection, the arithmetic mean interval of 54 days between radiological suspicion of recurrence and surgery was used as a predefined cut-off. This approach was chosen to reflect the overall distribution of surgical timing within the cohort and to ensure adequate group sizes for statistical comparison. We also used the median of 24 days as an alternative threshold in a second analysis to attain a more balanced group allocation.

### Tumor volumetry

Volumetric analysis of CE tumor volumes on preoperative and postoperative post-contrast T1-weighted MR was performed by semi-automated segmentation using the Brainlab™ software SmartBrush version 4.5 (Brainlab, Germany). The RANO Resect criteria were utilized to classify patients into RANO classes 1 to 3 [13] or the 0.175 ml threshold introduced by Stummer et al. [28]. Tumors with anatomical localization within motor, sensory, language and visual cortical and subcortical areas as described by Chang et al. [29], were defined as “eloquent”, in addition to deep-seated lesions (thalamic, basal ganglia).

### Study endpoints

Patients were followed until death or loss to follow-up (censored on the day of last follow-up), with survival after suspicion of recurrence defined as the interval from the date of radiological suspicion of recurrence to death from any cause or censorship. Progression-free survival 2 (PFS-2) was defined as the interval between repeat resection and the next radiological progression. ‘Transient’ postoperative neurological deficits were defined as deficits resolving within 30 days, which otherwise are termed ‘permanent’.

### Statistical analysis

Statistical analyses were performed using GraphPad PRISM (Version 10, GraphPad Software, Inc., La Jolla, USA). Continuous variables were reported as means ± standard deviation (SD) and/or median and interquartile range (IQR). Categorial variables were presented as numbers and percentages and compared using Chi-Square and Fisher’s exact test.

### Ethics approval

The Ethics Committee at the University of Heidelberg approved this retrospective analysis under S-455/2023 which has been performed in accordance with the ethical standards laid down in the 1964 Declaration of Helsinki and its later amendments and for which patient consent was not required.

## Results

### Time-course patterns of recurrence and subsequent repeat resection

This cohort of 150 GB patients with radiological suspicion of tumor recurrence experienced a heterogeneous management that always led to repeat resection. While upfront repeat resection was performed in just over half of cases (80 patients, 53%), salvage non-surgical therapy, including chemotherapy, immunotherapy and/or radiotherapy, was applied in 44 patients (29%), following a multidisciplinary tumor board (TB) recommendation. A watch-and-wait strategy was pursued in 20 patients (13%) when pseudo-progression could not be ruled out. In a minority of cases, patient preference led to deferral of the otherwise recommended repeat resection (6 patients, 4%; Table 1). In total, 44 patients initially received non-surgical treatment for a first radiological recurrence and subsequently underwent surgery following nonresponse and hence progression of recurrence in contrast-enhancing volume following an initial treatment-associated reduction.

**Table 1:** Time course until repeat resection at suspicion of recurrence of GB

Fate of patients with suspected tumor recurrence	n = 150%)
Decision for upfront repeat resection	80 (53.3)
Deferred Resection: Combination of systemic/radiotherapy	44 (29.3)^a^
CCNU/VP-16 = Lomustine/Etoposide	19
VXM01 plus avelumab	8
Parvovirus	7
Temozolomide	4
Bevacizumab	4
Irradiation	5
Other	3
Deferred Resection: Watch-and-wait^b^	20 (13.3)
Deferred Resection: Patient preference	6 (4)

Because patients received combination therapies, the subtotal exceeds 44. ^b^Suspicion of radiation necrosis. Summiert sich nicht zu 100%

**Fig. 1:**
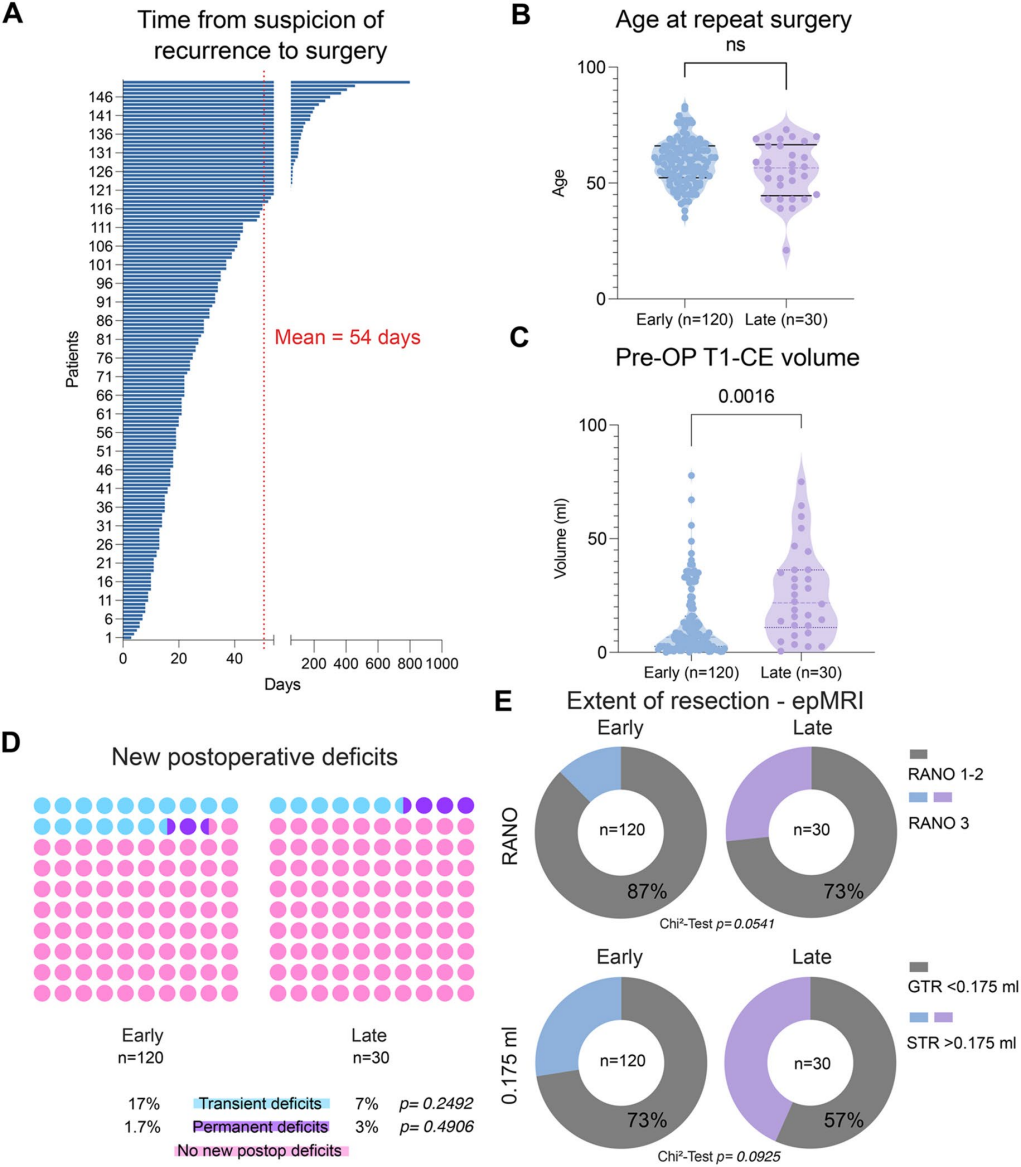

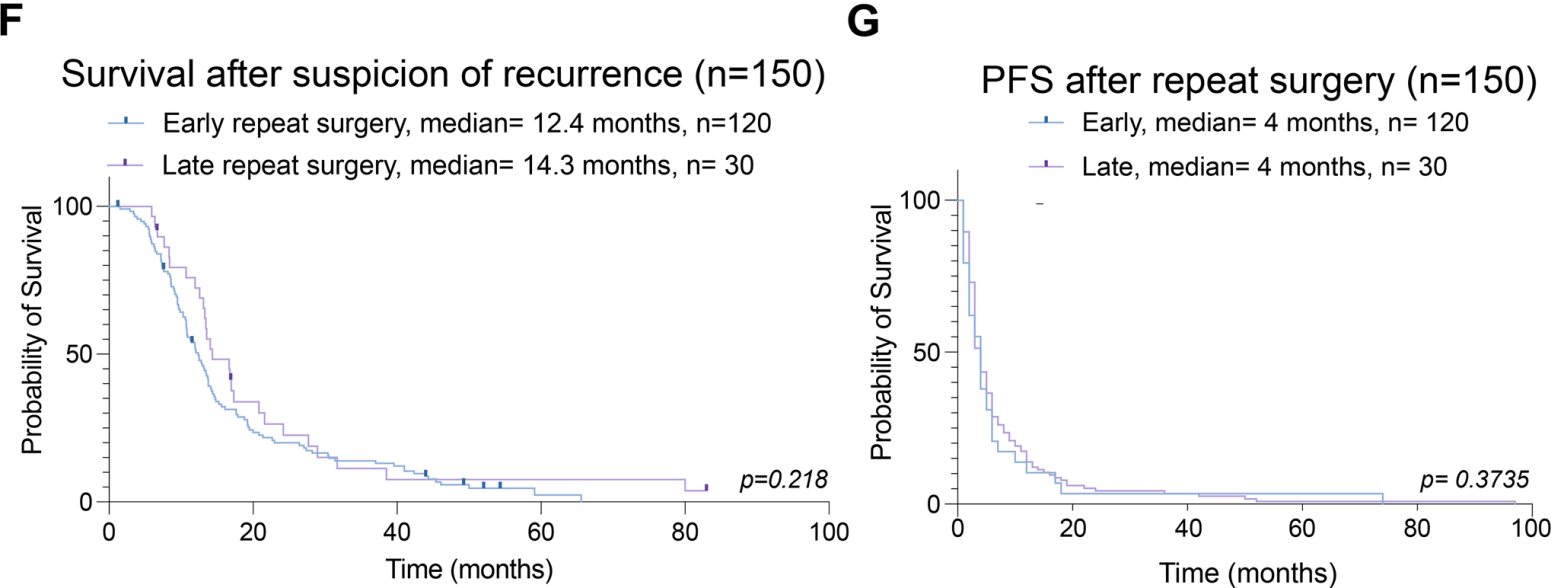
Time to repeat resection in patients with recurrent glioblastoma and comparison of clinical, radiological, and outcome parameters between early and late repeat resection groups by stratification based on mean time to surgery. **A**: Distribution of time from recurrence to repeat resection for the entire cohort (*n* = 150) demonstrates marked variability, with a mean interval of 54 days. **B**: Age at repeat resection was comparable between patients undergoing early (*n* = 120) and late (*n* = 30) repeat resection, with no statistically significant difference observed (ns, Welch’s test). **C**: Preoperative contrast-enhancing tumor volume on T1-weighted MRI differed between early and late repeat resection groups (*p* = 0.0016, Welch’s test). **D**: New postoperative neurological deficits after repeat resection were infrequent and did not differ significantly between early and late groups, including transient deficits (*p* = 0.2492, Chi-Square test) and permanent deficits (*p* = 0.4906, Fisher’s exact test). **E**: The extent of resection assessed on early postoperative MRI showed no statistically significant difference between early and late repeat resection when categorized according to RANO criteria (*p* = 0.0541, Chi-Square test) or when stratified by residual contrast-enhancing tumor volume using a cutoff of 0.175 ml (*p* = 0.0925, Chi-Square test). **F**: Overall survival after suspicion of recurrence did not differ significantly between early (median 12.4 months) and late (median 14.3 months) repeat resection groups (log-rank *p* = 0.218, Logrank [Mantel–Cox] test, censored patients = 10: Early group: 7, late 3). **G**: Progression-free survival after repeat resection was similar between early and late groups (median 4 months in both), with no significant difference observed (log-rank *p* = 0.3735, Logrank [Mantel–Cox] test))

All patients ultimately underwent repeat resection, with the interval between radiological suspicion of tumor recurrence and surgery varying widely, ranging from 3 to 799 days (≈26.6 months), with a median of 24 days and a mean of 54 days (SD = 80.4). Analysis of the cumulative interval distribution (Fig. 1A) revealed that 25% of repeat surgeries occurred within 18 days, and 75% occurred within 42 days (6 weeks) of suspicion of recurrence. For stratification purposes of what is regarded as an ‘early’ or ‘late’ surgery at suspicion of recurrence, we intially utilized the mean interval of 54 days as the threshold to distinguish early (≤54 days, *n* = 120) from late (>54 days, *n* = 30) repeat resection. Overall, the mean interval between initial resection and repeat resection was 430 (SD = 365) days.

### Comparison of clinical characteristics between early and late repeat resection patients

The early and late surgery groups demonstrated comparable baseline demographics. Age distribution was similar between early (mean 59.2 ± 9.73 years) and late (mean 55.4 ± 12.2 years) groups (*p* = 0.123, Welch’s test, Fig. 1B). Sex distribution showed a male predominance in both groups (early: 65% male; late: 53% male), with no statistically significant difference (*p* = 0.237, Chi-squared test). Tumor localization patterns were largely similar, with temporal location being most common in both early (44%) and late (33%) groups, followed by frontal location (28 and 30%, respectively). However, a notable exception was observed in insular tumors, which were significantly overrepresented in the late surgery group (0% vs 10%, *p* = 0.0074, Chi-Squared test). Tumor lateralization (right vs left) did not differ significantly between groups (*p* = 0.288, Chi-squared test). Karnofsky Performance Status (KPS) as a surrogate for preoperative functional status showed a non-significant higher score in the early group (median KPS 90) compared to the late group (median KPS 80, *p* = 0.085, Mann-Whitney test). The frequency of focal neurological deficits (FNDs) before repeat resection was similar (early: 59%; late: 60%; *p* = 0.9338, Chi-square test). Moreover, tumor eloquence was comparable between groups (early: 45%; late: 47%; *p* = 0.8697, Chi-square test) and consequently the application of intraoperative neuromonitoring (IONM) and awake craniotomy (early: 9%; late: 13%; *p* = 0.759, Chi-square test). Notably, preoperative CE tumor volumes were significantly lower in the early group (mean 12.7 ± 14.9 ml) compared with the late group (mean 25.86 ± 19.8 ml; *p* = 0.0016, Welch’s t-test, Fig. 1C).

### Differences in extent of resection and functional outcomes between early and late repeat resection patients based on a mean cut-off of 54 days

Interestingly, a subtle difference in post-operative RTV was noticed between the early and the late surgery groups (mean early group = 0.53 ± 1.50 ml vs late group = 1.58 ± 2.93 ml), without reaching statistical significance (*p* = 0.066, Welch’s t-test, Table 2). Because a T1-CE RTV mean of >1 ml would have an implication for the RANO Resect classification, we classified patients according to RANO Resect criteria (RANO 1 and 2 vs RANO 3) based on RTV accordingly, revealing a non-significant overrepresentation of RANO 3 resections in the late surgery group (27% vs 73% RANO 1 and 2) compared to the early group (13% vs 87% RANO 1 and 2, *p* = 0.054, Chi-square test). When stratifying by gross total resection (GTR) versus subtotal resection (STR) using the 0.175 ml threshold set by Stummer et al. [28], the late group also demonstrated a non-significantly lower proportion of GTR (RTV < 0.175 ml, 57% vs 73% in the early group, *p* = 0.093, Chi-square test), suggesting that delayed recurrences may not have achieved the desired EOR. Regarding functional outcomes, post-operative KPS at discharge was consistent across both groups (median KPS 80 for both; *p* = 0.228, Mann-Whitney test). Corroborating this, we examined the rate of transient and permanent post-operative neurological deterioration and found non-significant differences in rates of transient deficits between the early and late groups (17% vs 7% *p* = 0.249, Chi-square test) and permanent deficits (1.7% vs 3%, *p* = 0.491, Chi-square test), indicating that the timing of repeat resection did not substantially affect the risk of neurosurgical morbidity. The overall rate of post-operative transient and permanent deterioration in this cohort was 15 and 2%, respectively. The MGMT promoter methylation status showed similar distribution between early and late surgery groups: Approximately half the patients had non-methylated MGMT status in both groups (early: 51%; late: 57%), with methylated status present in approximately 30% of each group (*p* = 0.9118, Chi-square test). After repeat resection, adjuvant treatment patterns revealed several significant differences between groups. CCNU/VP-16 combination therapy was significantly more common in the early surgery group (53% vs 43%, *p* = 0.0224, Chi-square test), probably due to such options regarded as redundant in patients of the late group previously exposed to this regimen. Accordingly, patients in the late surgery group were significantly more likely to receive no adjuvant treatment following reoperation (33% vs 16%, *p* = 0.0299). Postoperative radiotherapy was administered in 26 patients (17%), including 19 patients in the early group (16%) and 7 patients in the late group (23%), with no significant difference between groups (*p* = 0.332, Chi-square test). Other treatment modalities, including temozolomide and bevacizumab, showed no significant differences between groups. Survival analysis revealed no significant difference in overall survival from suspicion of recurrence to death or censorship between early (12.4 months) and late groups (14.3 months, *p* = 0.218, Logrank [Mantel–Cox] test, Fig. 1F, patients censored = 10). Analysis of PFS-2 similarly showed no significant difference between groups, with a median PFS-2 of 4 months in both groups (*p* = 0.372, Logrank [Mantel–Cox] test, Fig. 1F). Altogether, these data indicate that EOR and adjuvant treatment strategies might vary depending on the timepoint of surgery but did not significantly impact overall survival or progression-free survival after repeat resection.

**Table 2:** Comparative analysis for baseline characteristic, treatment modalities and peri-operative functional status between patients receiving early and late resection for recurrent GB, applying the mean interval of 54 days between radiological suspicion of recurrence until repeat resection

Variable	All (n = 150)	Early (n = 120)	Late (n = 30)	p-value
Resection				<0.0001^a^
Upfront	80	80 (53)	0	
Deferred	70	40 (27)	30 (20)	
Sex				
Male	94 (63%)	78 (65%)	16 (53%)	0.237^a^
Female	56 (37%)	42 (35%)	14 (47%)	
Mean Age (SD)	58.4 (10.4)	59.2 (9.73)	55.4 (12.2)	0.123^d^
Tumor localization				
Frontal	43 (28%)	34 (28%)	9 (30%)	0.857^a^
Temporal	63 (42%)	53 (44%)	10 (33%)	0.282^a^
Parietal	24 (16%)	20 (17%)	4 (13%)	0.656^a^
Occipital	16 (11%)	13 (11%)	3 (10%)	0.895^a^
Insular	3 (2%)	0 (0%)	3 (10%)	0.007^b^
Deep	1 (<1%)	0 (0%)	1 (3%)	0.200^b^
Tumor lateralization				0.288^a^
Right	77 (51%)	59 (49%)	18 (60%)	
Left	73 (49%)	61 (51%)	12 (40%)	
Tumor eloquence	68 (45%)	54 (45%)	14 (47%)	0.870^a^
Median KPS before surgery	80	90	80	0.085^c^
T1-CE volume on preMRI	15.4 (16.7)	12.7 (14.9)	25.9 (19.8)	0.002^d^
IONM ±awake craniotomy	18 (12%)	14 (9%)	4 (13%)	0.759^b^
T1-CE RTV on epMRI	0.74 (1.91)	0.53 (1.50)	1.58 (2.93)	0.066^d^
KPS at discharge (median)	80	80	80	0.228^c^
MGMT promoter methylation status				0.911^a^
Methylated	43 (29%)	34 (28%)	9 (30%)	
Non-methylated	78 (52%)	61 (51%)	17 (57%)	
Unknown	29 (19%)	25 (21%)	4 (13%)	
Adjuvant therapy after repeat resection				
Temozolomide	24 (16%)	21 (18%)	3 (10%)	0.412^b^
CCNU/VP-16	77 (51%)	64 (53%)	13 (43%)	0.022^a^
BCNU/VM-26	9 (6%)	8 (7%)	1 (3%)	0.688^b^
Other agents:				
Bevacizumab	15 (10%)	12 (10%)	3 (30%)	>0.999^b^
Other	19 (13%)	15 (13%)	4 (13%)	>0.999^b^
Unknown	8 (5%)	7 (6%)	1 (3%)	>0.999^b^
Radiotherapy	26 (17%)	19 (16%)	7 (23%)	0.332^a^
No adjuvant treatment	29 (19%)	19 (16%)	10 (33%)	0.030^a^

SD = Standard deviation; FND = Focal Neurological deficit; KPS = Karnofsky Performance Scale; CE = Contrast enhancing; preMRI = preoperative MRI scan; IONM = intraoperative neuromonitoring; RTV = Residual tumor volume; epMRI = early postoperative MRI; MGMT = O6-methylguanine-DNA methyltransferase. CCNU/VP-16 = Lomustine/Etoposide ^a^Chi-square test, ^b^Fisher exact, ^c^Mann-Whitney test, ^d^Welch’s test

### Timing of repeat resection and associated clinical, radiological, and outcome parameters using a median cut-off of 24 days between suspicion of recurrence and repeat surgery

**Fig. 2:**
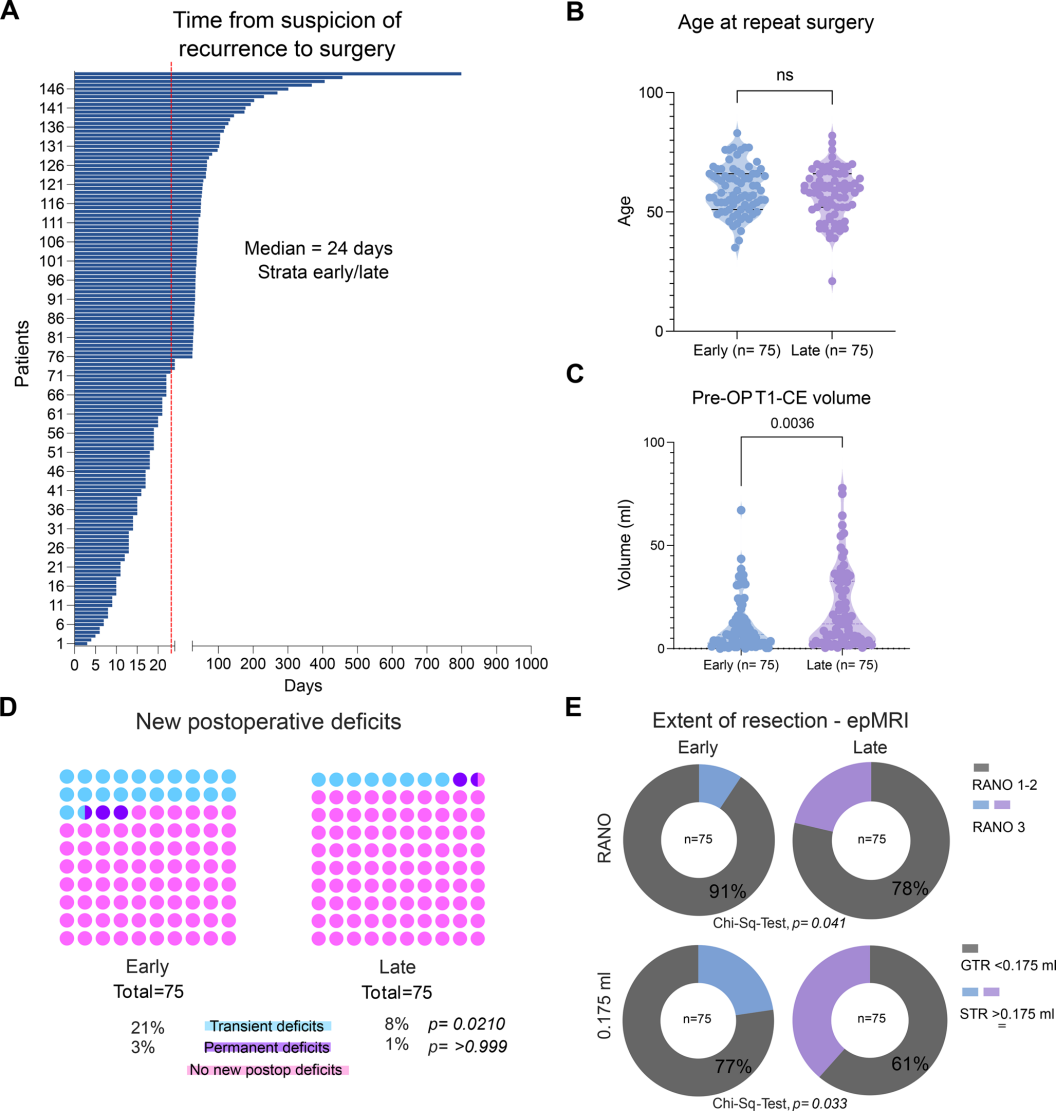

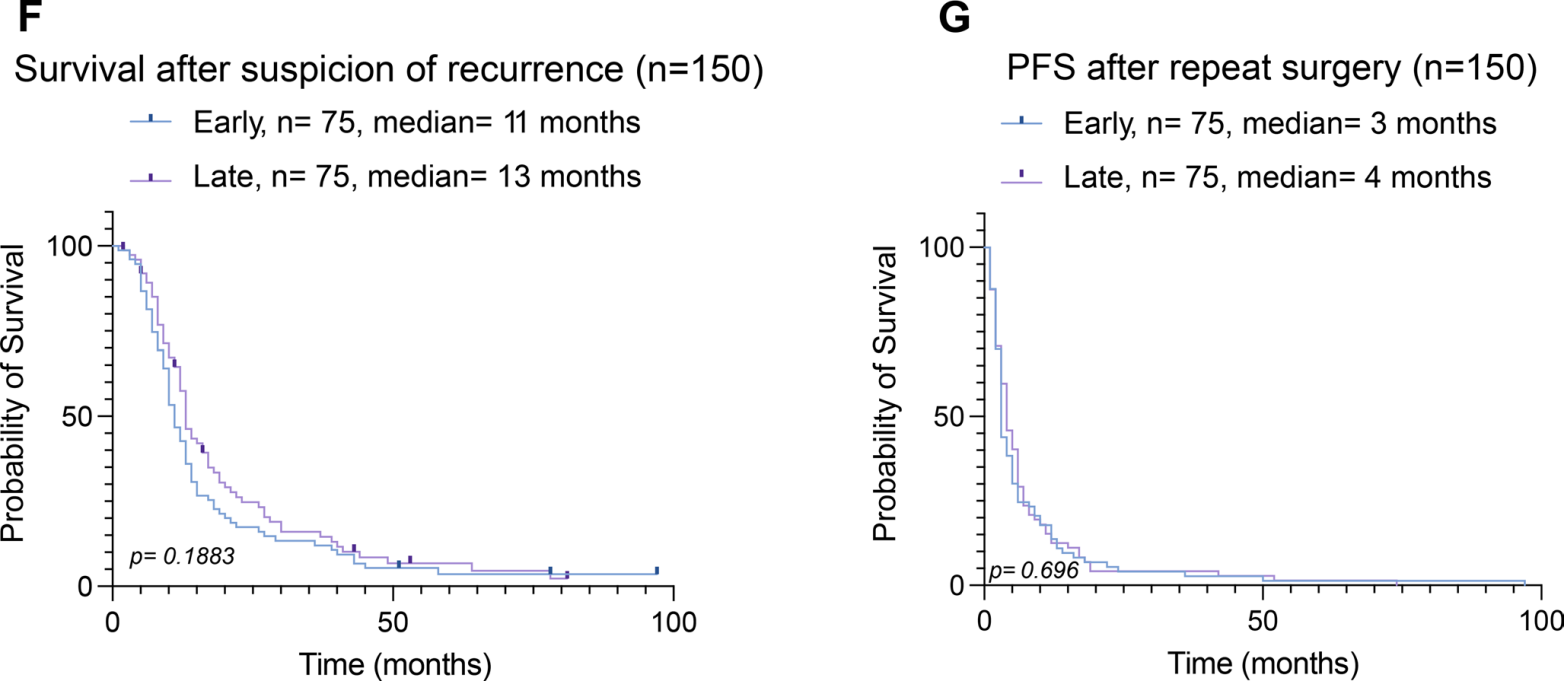
Time to repeat resection in patients with recurrent glioblastoma and comparison of clinical, radiological, and outcome parameters between early and late repeat resection groups by stratification based on median time to surgery. **A**: Distribution of time from recurrence to repeat resection for the entire cohort (*n* = 150) demonstrates marked variability, with a median interval of 24 days. **B**: Age at repeat resection was comparable between patients undergoing early (*n* = 75) and late (*n* = 75) repeat resection, with no statistically significant difference observed (ns, Welch’s test). **C**: Preoperative contrast-enhancing tumor volume on T1-weighted MRI differed between early and late repeat resection groups (*p* = 0.0036, Welch’s test). **D**: New postoperative neurological deficits after repeat resection were infrequent and were overrepresented in the early group with transient deficits being significantly overrepresented (*p* = 0.0210, Chi-Square test) and permanent deficits (*p* > 0.999, Fisher’s exact test). **E**: The extent of resection assessed on early postoperative MRI showed statistically significant differences between early and late repeat resection when categorized according to RANO criteria (*p* = 0,041, Chi-Square test) or when stratified by residual contrast-enhancing tumor volume using a cutoff of 0.175 ml (*p* = 0.033, Chi-Square test). **F**: Overall survival after suspicion of recurrence did not differ significantly between early (median 11 months) and late (median 13 months) repeat resection groups (*p* = 0.188, Logrank [Mantel–Cox] test, censored patients = 10: Early group: 7, late 3). **G**: Progression-free survival after repeat resection was similar between early and late groups (median 3 months in early and 4 months in late), with no significant difference observed (*p* = 0.696, Logrank [Mantel–Cox] test)

When stratifying by the mean interval of 54 days between radiological suspicion of recurrence and repeat resection, we observed no significant time-dependent oncological and functional differences; however, due to a right-skewed patient distribution, most patients assigned to the early surgery group. To account for potential differences attributable to group size, we also used the median interval of 24 days as a cutoff, with patients stratified into an early (≤24 days, *n* = 75) and a late (>24 days, *n* = 75) repeat resection group (Fig. 2A).

Baseline demographic characteristics (age at the time of repeat resection; sex distribution) were comparable between groups (Table 2; Fig. 2B). In contrast, preoperative T1-CE tumor volumes differed significantly between groups, with patients in the late surgery group presenting with larger tumor volumes at the time of surgery (*p* = 0.0036, Welch’s t-test; Fig. 2C). However, new postoperative neurological deficits were significantly overrepresented in the early surgery group, driven by a significantly higher rate of transient neurological deficits (*p* = 0.021, Chi-square test), while permanent neurological deficits were rare and did not differ between groups (*p* > 0.999, Fisher’s exact test; Fig. 2D). Assessment of the EOR on epMRI revealed significant differences between early and late surgery groups. When categorized according to RANO Resect criteria, the EOR differed significantly between groups (*p* = 0.041, Chi-square test; Fig. 2E). Similarly, stratification based on residual CE tumor volume using a cutoff of 0.175 ml demonstrated significant differences between early and late surgery patients (*p* = 0.033, Chi-square test), indicating a higher likelihood of more extensive resections in the early surgery group. Despite these differences in preoperative tumor burden, postoperative neurological deficits, and EOR, survival outcomes were comparable. Overall survival from suspicion of recurrence did not differ significantly between groups, with median overall survival of 11 months in the early and 13 months in the late group (log-rank *p* = 0.188, Logrank [Mantel–Cox] test; censored patients = 10; Fig. 2F). Moreover, PFS-2 was comparable, with a median of 3 months in the early and 4 months in the late group (log-rank *p* = 0.696, Logrank [Mantel–Cox] test; Fig. 2G). Also under this stratification, patients in the late surgery group (>24 days) were significantly less likely to receive adjuvant treatment following reoperation (33% vs 16%, *p* = 0.0299). Interestingly, patients in the late surgery group were significantly less likely to receive adjuvant temozolomide treatment following reoperation (23% vs 9%, *p* = 0.026) but more likely to undergo postoperative irradiation (early 9% and late 25%, *p* = 0.0096, Table 3). Taken together, in comparison to using the mean interval as a stratification threshold, application of the median cut-off resulted in a more balanced group allocation and accentuated differences in perioperative parameters such as preoperative tumor volume, EOR, and transient postoperative neurological deficits. However, irrespective of the chosen cut-off (mean of 54 days or median of 24 days), survival outcomes remained comparable between early and late repeat resection groups.

**Table 3:** Comparative analysis for baseline characteristic, treatment modalities and peri-operative functional status between patients receiving early and late resection for recurrent GB, applying the median interval of 24 days between radiological suspicion of recurrence until repeat resection

Variable	Early (n = 75)	Late (n = 75)	p-value
Resection			<0.0001^a^
Upfront	75 (50)	5 (3)	
Deferred	0	70 (47)	
Gender			0.736^a^
Male	48 (64%)	46 (61%)	
Female	27 (36%)	29 (39%)	
Mean age (SD)	59.2 (10.1)	57.6 (10.6)	0.330^d^
MGMT promoter methylation status			0.615^a^
Methylated	23 (31%)	20(27%)	
Non-methylated	38 (51%)	40(53%)	
Unknown	14 (18%)	15 (20%)	
Tumor localization			
Frontal	22 (29%)	21 (28%)	0.857^a^
Temporal	31 (41%)	32 (43%)	0.869^a^
Parietal	14 (19%)	10 (13%)	0.373^a^
Occipital	8 (11%)	8 (11%)	>0.999^a^
Insular	0%)	3 (4%)	0.120^b^
Deep	0 (0%)	1 (1%)	>0.999^b^
Lateralization			0.624^a^
Right	40 (53%)	37 (49%)	
Left	35 (47%)	38 (51%)	
Tumor eloquence	34%)	34%)	>0.999^a^
T1-CE volume on preMRI	11.4 (12.9)	19.3 (19.1)	0.004^d^
IONM ± awake craniotomy	9 (12%)	9 (12%)	>0.999^a^
Median KPS before surgery	90	80	0.981^c^
Median KPS at discharge	80	80	0.471^c^
Adjuvant therapy after re-resection			
Temozolomide	17 (23%)	7 (9%)	0.026^a^
CCNU/VP-16	39 (52%)	38 (51%)	0.870^a^
BCNU/VM-26	7 (9%)	2 (3%)	0.166^b^
Unknown	3 (4%)	5 (7%)	0.719^b^
Other agents:			
Bevacizumab	8 (11%)	7 (9%)	0.786^a^
Other	11 (15%)	8 (11%)	0.461^a^
Radiotherapy	7 (9%)	19 (25%)	0.0096^a^
No adjuvant treatment	11 (15%)	18 (24%)	0.148^a^

SD = Standard deviation; FND = Focal Neurological deficit; KPS = Karnofsky Performance Scale; CE = Contrast enhancing; preMRI = preoperative MRI scan; IONM = intraoperative neuromonitoring; RTV = Residual tumor volume; epMRI = early postoperative MRI; MGMT = O6-methylguanine-DNA methyltransferase. CCNU/VP-16 = Lomustine/Etoposide ^a^Chi-square test, ^b^Fisher exact, ^c^Mann-Whitney test, ^d^Welch’s test

## Discussion

While there is increasing evidence supporting the benefit of repeat resection in the multimodal treatment of rGB, the optimal timing for surgery remains unclear. In this cohort, tumors resected at later time points, i.e. approximately two months after radiological suspicion of recurrence, were significantly larger at the preoperative stage than tumors resected at earlier time-points. Despite this, only subtle, non-significant differences in EOR were observed between both groups, with a trend toward higher RTVs and hence lower rates of GTR in the late surgery group. However, these differences did not translate into measurable differences in overall or progression-free survival after repeat resection. In addition, a longer interval between radiological suspicion of recurrence and repeat resection was not associated with an increased risk of transient or permanent postoperative neurological deterioration. Notably, these differences narrowly failed to reach statistical significance, which may in part be attributable to a right-skewed distribution of the analysis when applying the mean interval of 54 days from radiological suspicion of recurrence until repeat resection as a cut-off, resulting in a higher proportion of patients in the early surgery group. A larger sample size or a greater representation of patients undergoing surgery at later time points might therefore render these differences statistically significant.

Beyond previous studies showing that early recurrence and hence a short time-span between primary and repeat resection in GB is associated with poor prognosis [30], there is yet no understanding of how differences in patient management upon suspicion of tumor recurrence can influence or are influenced by surgical decision-making. For example, it is conceivable that while localization of non-eloquent brain regions showed no difference between the early and late repeat resection cohort, a notable exception was observed in insular tumors, which were significantly overrepresented in the late group (10% vs 0%). This reflects surgery-reluctant decision-making when anatomically challenging regions are involved to avoid the eventuality of neurosurgical morbidity, with eloquent location known to be associated with reduced survival in rGB [31, 32].

These findings highlight the considerable heterogeneity and uncertainty that characterize clinical decision-making in patients with suspected recurrent glioblastoma. While upfront repeat resection was pursued in just over half of cases, a substantial proportion of patients were initially managed with non-surgical treatment or close radiological follow-up, which also reflects the challenge of distinguishing true tumor recurrence from treatment-related changes [33, 34]. This intermediate clinical state—where surgery is deferred to allow for assessment of response to systemic therapy or to monitor disease evolution—represents a quite occasional but rather poorly defined scenario in routine practice.

Like studies on primary GB, differences in tumor volume between early and late repeat resection groups demonstrate volumetric tumor growth during extended observation intervals [35, 36]. The diverse distribution of the “time-to-surgery” intervals indicates that while most patients underwent early repeat resection, a distinct subset experienced prolonged disease control before requiring repeat surgical intervention [31, 37].

But what defines a “delayed” surgery in the context of recurrent GB? Given the highly skewed distribution of the interval between radiological suspicion of recurrence and repeat resection, the mean interval of 54 days was not intended to represent an optimal or biologically meaningful cut-off. Rather, it was used as a pragmatic threshold to distinguish patients undergoing repeat resection within a relatively short interval from those experiencing clearly prolonged decision pathways, often involving salvage therapy, surveillance, or deferred surgery.

Interestingly, re-analysis using the median interval of 24 days yielding balanced subgroups mostly confirmed the findings observed under the 54-day stratification. Under both cut-offs, delayed repeat resection was associated with larger preoperative tumor volumes, less favorable extent of resection, and differences in postoperative treatment patterns, while overall survival after suspicion of recurrence and progression-free survival after repeat surgery remained comparable between early and late surgery groups under all stratifications. Importantly, postoperative neurological morbidity was low across all analyses. Although transient neurological deficits were more frequently observed in the early surgery group under the 24-day stratification, permanent neurological deficits were rare and did not differ between groups, consistent with findings from the 54-day cut-off. Collectively, these results suggest that while the timing of repeat resection influences surgical and treatment-related parameters, it does not materially affect survival outcomes or long-term functional outcome. This reflects both the heterogeneity of patients involved and the attenuated influence of the burden of contrast-enhancing tumor at this stage of the disease. Future studies using time-to-surgery as a continuous variable or employing time-dependent modeling will be required to further refine the relationship between surgical timing and outcome.

In our study, patients with rGB conferred a median survival after suspicion of recurrence of 12 months in the early group and 14 months in the late group without significant differences, possibly owing to an overall low sample number. This is consistent with survival outcomes reported in previous trials focusing on GB patients with 1^st^ repeat resection, which range at around one year [9, 11]. In addition, progression-free survival is known to correlate with response to treatment and in-turn to correlate with survival [38]. Indeed, there were no differences pertaining to PFS-2 between early and late repeat resection patients in this study.

A potential advantage of ‘early’ repeat resection lies in the opportunity to obtain tumor tissue for molecularly-guided treatment [15, 39–44], enabling personalized therapeutic approaches [45, 46], like mTOR-targeted therapy [47, 48]. Such strategies are more likely to be implemented at an earlier disease stage, when patients are less heavily pretreated and therefore more amenable to therapy. This concept is supported by the observed differences in post-resection adjuvant therapy patterns between groups in our study. Notably, patients undergoing early repeat resection more frequently received chemotherapy (CCNU/VP-16) compared with those in the late group. This may reflect a greater availability of effective systemic treatment options in earlier disease stages, whereas similar regimens may be regarded as redundant or less synergistic in patients who have already been exposed to different lines of therapy. In line with this interpretation, patients in the late surgery group were significantly more likely to receive no adjuvant treatment following reoperation, suggesting a narrowing of therapeutic options and potentially diminished treatment tolerance at later time points. Together, these findings underscore the potential role of early surgical intervention not only in effective cytoreduction but also as a facilitator of subsequent, more active adjuvant treatment strategies.

An additional advantage of early repeat resection is increased diagnostic certainty, since tissue sampling usually allows reliable exclusion of radiation necrosis. At present, no imaging modality can fully replace histological confirmation, and advanced techniques such as PET imaging were not routinely used in our cohort [49]. Even though we selected patients with confirmed histopathological diagnosis of rGB, advances in artificial intelligence enabling pixel-level analysis and the integration of multi-sequence and multi-modality data show considerable promise for distinguishing treatment-related effects from recurrent tumor [50], which would help improve patient stratification for individualizing decision-making.

The overall rate of transient and permanent postoperative neurological deficits in our cohort was 15% and 2% respectively, with no significant difference observed between the early and late surgery groups. This is lower than what was reported in the literature for similar cohorts (surgical complication rates 8% to 30%) [9, 51, 52], as it may reflect evovling advances in intraoperative neuromonitoing whileand challenging the notion that patients with recurrent glioblastoma are at a higher risk of surgical and neurological complications [53].

This study has several limitations, especially given the retrospective study design. This study does not address whether salvage therapy prior to repeat resection affects tumor growth to off-set a potential benefit of an otherwise early surgery. We also acknowledge that the approach of using the mean “time-to-surgery” interval of 54 days results in a smaller and more heterogeneous late surgery subgroup and may limit statistical power; however, it reflects the inherent heterogeneity of recurrent glioblastoma management and allows clinically interpretable group comparisons aligned with routine practice. However, this study was conceived to examine the effect of multi-disciplinary decision-making on the longitudinal neurosurgical management of patients with recurrent glioblastoma rather than to explore differences between different therapies. Also, different growth dynamics were not taken into consideration, as this might have influenced the rationale of surgery to prolong survival or alleviate symptoms through cytoreduction [54]. This study also does not examine the effect of these interventions on the quality of life or the cognitive performance of affected patients, especially since higher disease burden and cognitive impairment are known to reduce patient survival in glioblastoma [55, 56].

Nevertheless, these findings suggest that while deferred surgery may risk continued tumor growth, a carefully selected watch-and-wait strategy or pre-surgical salvage therapy does not compromise functional safety or oncological outcomes in patients undergoing repeat resection for recurrent glioblastoma.

## Conclusion

Tumor resection of recurrent glioblastoma beyond two months after radiological suspicion of recurrence was associated with non-significant differences in the EOR compared to resection at an earlier time point, without a statistically significant difference in patient outcome. Importantly, delaying surgery was not associated with an increased risk of postoperative neurological deterioration. These findings suggest that delaying repeat surgery for selected patients could be justified, especially if pseudoprogression cannot be ruled out or systemic salvage therapy has not been assessed for efficacy in individual patients.

## Data Availability

The datasets generated during and/or analyzed during the current study are available from the corresponding author on reasonable request.
